# Chrononutrition and Cardiometabolic Health: An Overview of Epidemiological Evidence and Key Future Research Directions

**DOI:** 10.3390/nu16142332

**Published:** 2024-07-19

**Authors:** Oluwatimilehin E. Raji, Esther B. Kyeremah, Dorothy D. Sears, Marie-Pierre St-Onge, Nour Makarem

**Affiliations:** 1 Department of Epidemiology, Mailman School of Public Health, Columbia University Irving Medical Center, New York, NY 10032, USA; oer2105@cumc.columbia.edu (O.E.R.); ebk2150@cumc.columbia.edu (E.B.K.); 2 College of Health Solutions, Arizona State University, Tempe, AZ 85287, USA; dorothy.sears@asu.edu; 3 Department of Medicine, University of California San Diego School of Medicine, La Jolla, CA 92093, USA; 4 Department of Family Medicine, University of California San Diego School of Medicine, La Jolla, CA 92093, USA; 5 Center for Circadian Biology, University of California San Diego, La Jolla, CA 92093, USA; 6 Department of Medicine, Columbia University Irving Medical Center, New York, NY 10032, USA; ms2554@cumc.columbia.edu; 7 Sleep Center of Excellence, Columbia University Irving Medical Center, New York, NY 10032, USA

**Keywords:** chrononutrition, temporal eating patterns, circadian rhythms, eating timing, eating regularity, cardiometabolic health, cardiovascular health, cardiovascular disease prevention

## Abstract

Chrononutrition is a rapidly evolving field of nutritional epidemiology that addresses the complex relationship between temporal eating patterns, circadian rhythms, and metabolic health, but most prior research has focused on the cardiometabolic consequences of time-restricted feeding and intermittent fasting. The purpose of this topical review is to summarize epidemiological evidence from observational and intervention studies regarding the role of chrononutrition metrics related to eating timing and regularity in cardiometabolic health preservation and cardiovascular disease prevention. Observational studies are limited due to the lack of time-stamped diet data in most population-based studies. Findings from cohort studies generally indicate that breakfast skipping or the later timing of the first eating occasion, a later lunch and dinner, and a greater proportion of caloric intake consumed in the evening are associated with adverse cardiometabolic outcomes, including higher risk for coronary heart disease, hypertension, type 2 diabetes, obesity, dyslipidemia, and systemic inflammation. Randomized controlled trials are also limited, as most in the field of chrononutrition focus on the cardiometabolic consequences of time-restricted feeding. Overall, interventions that shift eating timing patterns to earlier in the day and that restrict evening caloric intake tend to have protective effects on cardiometabolic health, but small sample sizes and short follow-up are notable limitations. Innovation in dietary assessment approaches, to develop low-cost validated tools with acceptable participant burden that reliably capture chrononutrition metrics, is needed for advancing observational evidence. Culturally responsive pragmatic intervention studies with sufficiently large and representative samples are needed to understand the impact of fixed and earlier eating timing schedules on cardiometabolic health. Additional research is warranted to understand the modifiable determinants of temporal eating patterns, to investigate the role of chrononutrition in the context of other dimensions of diet (quantity, quality, and food and nutrition security) in achieving cardiometabolic health equity, and to elucidate underlying physiological mechanisms.

## 1. Introduction

Chrononutrition is a rapidly evolving field of nutritional epidemiology that addresses the complex relationship between temporal eating patterns, circadian rhythms, and metabolic health to elucidate how eating timing metrics influence health preservation and chronic disease risk [1,2,3]. The timing of food intake relative to circadian rhythms, representing the innate 24 h cycles in human behavior, physiology, and metabolism, may be particularly important for preserving cardiometabolic health (CMH) and preventing cardiovascular disease (CVD). While light exposure is the predominant zeitgeber, food intake also entrains the circadian system, and research shows that metabolic health is influenced not just by the quantity and quality of dietary intake, but also by the timing, regularity, and energy distribution of eating occasions in the 24 h day and across days [3,4]. Indeed, aspects of chrononutrition, stemming from environmental, cultural, and physiological factors as well as personal preference and lifestyle choices that alter eating behaviors, have been linked to the etiology of CVD, type 2 diabetes, metabolic syndrome, hypertension, and obesity [5].

Enhancing cardiometabolic disease prevention approaches with strategies grounded in chrononutrition could play a pivotal role in reducing the public health burden of these conditions [1]. Through targeted health education and promotion measures, individuals can be informed about the implications of eating timing on health and guided towards establishing and preserving consistent eating routines synchronous with their circadian rhythms. Such measures could potentially delay the emergence of metabolic risk factors linked to cardiometabolic and cardiovascular diseases or aid in their management. For example, “Life’s Essential 8”, a cardiovascular health construct developed by the American Heart Association (AHA) to guide primordial prevention efforts, includes diet as a metric [6]. However, this metric mainly addresses dietary quality and excludes dietary dimensions related to the timing of meals and other eating occasions. Screening for and addressing eating timing and regularity could potentially provide a more comprehensive assessment of nutritional health and unlock the true potential of dietary approaches in primordial prevention efforts aimed at CMH preservation.

Despite the immense promise of this field for addressing the chronic disease burden and persistent CMH inequities, many knowledge gaps remain. This topical review summarizes the epidemiologic evidence, encompassing both observational and intervention studies, on temporal eating patterns in relation to CMH. Given that multiple prior reviews have focused on time-restricted feeding and nightly fasting duration in relation to CMH [3,7,8], we focused this review on associations of eating timing, meal skipping, and eating timing regularity with cardiometabolic outcomes and CVD. We also highlight knowledge gaps and important research directions necessary to advance the field and translate chrononutrition research into public health policy and action.

## 2. Eating Timing and Regularity Patterns in the US and Other Populations

Despite the widely held view that breakfast holds paramount importance as the day’s initial meal, research indicates that 20% to 30% of US adults regularly omit this eating occasion [9]. Further, there has been a decline in the reported consumption of breakfast and lunch over the past 40 years [10]. Similar trends are observed in Asian populations; for instance, a multicenter Japanese study showed that ~23% of adults skipped breakfast [11], and ~45% of Korean adults do not report caloric intake during the morning hours [12]; this proportion is even higher among Korean office workers (~70%) who report skipping breakfast due to time constraints [13]. In the Mediterranean population, breakfast, lunch, and dinner are typically consumed later than in the US and other populations [14,15,16]. For example, lunch is consumed, on average, between 2:00 and 3:00 pm, compared to 1:00 and 2:00 pm in the US population [14,15]. Notably, within the framework of the Mediterranean diet, lunch holds particular importance, accounting for approximately 40% of the daily caloric consumption compared to about 24% in the US [17,18].

In terms of the distribution of calories across the 24 h day, data from a smartphone application show that US adults eat around the clock and that calorie consumption extends beyond typical meal hours [19]. There is also a tendency for later eating timing, as >75% of caloric intake occurs after noon with ~38% of calories consumed after 6:00 pm [19]. A tendency for substantial nighttime eating was also observed in the National Health and Nutrition Examination (NHANES) survey, whereby over a third of individuals reported consuming ≥25% of their daily caloric intake after 7:00 pm and ~10% reported consuming ≥50% of caloric intake after 7:00 pm or any calorie intake after 11:00 pm [20].

In addition to eating timing within the 24 h day, eating timing across days has emerged as a novel potential contributor to CMH [19,20,21,22]. The term ‘eating jet lag’ describes the difference in mealtimes between weekdays and weekends [21,22]. In a 2021 study examining racially and ethnically diverse US women, the timing of the first eating occasion was ~1.5 h later on weekends than on weekdays [22]. Similarly, breakfast timing was ~2 h later on weekends relative to weekdays in a study of young Spanish and Mexican adults [21]. In NHANES and the Continuing Survey of Food Intakes by Individuals, night eating (i.e., ≥25% kcal after 7:00 pm, ≥50% kcal after 7:00 pm, and any eating after 11:00 pm) was more common during the weekend than weekday [20]. Consistent with these findings, data from a smartphone app revealed that US adults have erratic eating timing patterns with substantial day-to-day variation, particularly for timing of the first eating occasion with later eating start times being observed on weekends compared to weekdays (10:26 vs. 09:21 am) [19].

Sociocultural, economic, and environmental factors can substantially influence when someone eats and could help to explain differences in temporal eating patterns observed across populations [23]. In Mediterranean cultures, lunch accounts for a substantial proportion of daily caloric intake, despite the later overall timing of lunches and dinners; whereas in the USA and in central and northern European countries, dinner and after dinner snacks tend to account for the largest percentage of daily caloric intake [10,16,23]. Further, the emphasis on breakfast consumption could vary across cultures with breakfast skipping predisposing to later eating timing and higher caloric intake in the evening and generally being linked to having a lower socioeconomic status [24,25]. Relatedly, being an essential worker and/or a shift worker is also associated with later and irregular eating timing patterns [25,26]. This can help to explain the higher prevalence of adverse cardiometabolic outcomes among essential workers and shift workers and among minoritized populations that tend to be overrepresented in these occupations. 

**Figure 1:**
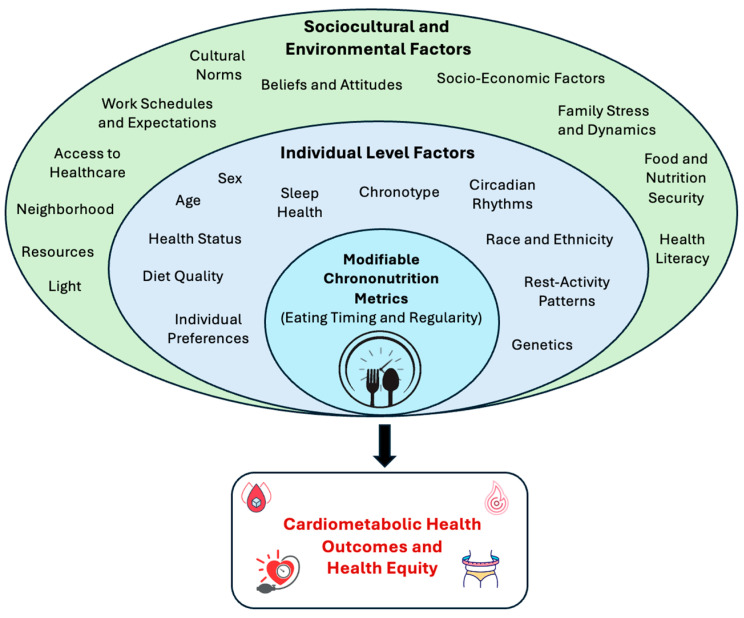
Determinants of eating timing and regularity.

Individual behavioral factors can also influence the timing of food intake, the most notable of which is sleep. Having a shorter sleep duration and going to bed later provides more opportunities for later eating timing; in addition, irregular sleep schedules are likely correlated with irregular eating schedules [23]. This is exacerbated by light exposure at night, which can delay sleep and extend eating periods later into the night. Other dietary dimensions can also influence meal timing; for instance, food insecurity can lead to less structured mealtimes and irregular eating timing patterns [27]. In addition, there is likely a bidirectional association between chrononutrition metrics and diet quality, with later eating times being linked to unhealthy food choices and exacerbating the adverse health effects of poor diet quality [28,29]. However, there is a dearth of evidence on the behavioral underpinnings of temporal eating patterns, particularly the influence of sleep health, physical activity, and other diet dimensions on when someone eats. Other individual-level characteristics such as age, sex, chronotype, genetic predisposition and health status could also shape eating timing patterns, with later and less structured eating times observed among those with later chronotypes (representing the behavioral manifestation of endogenous circadian rhythms), in younger age groups, and among individuals with obesity [23,30]. The multi-level predictors of chrononutrition metrics influencing cardiometabolic outcomes and the attainment of health equity and warranting further investigation are summarized in Figure 1.

## 3. Overview of Mechanisms Linking Temporal Eating Patterns to Cardiometabolic Health

**Figure 2:**
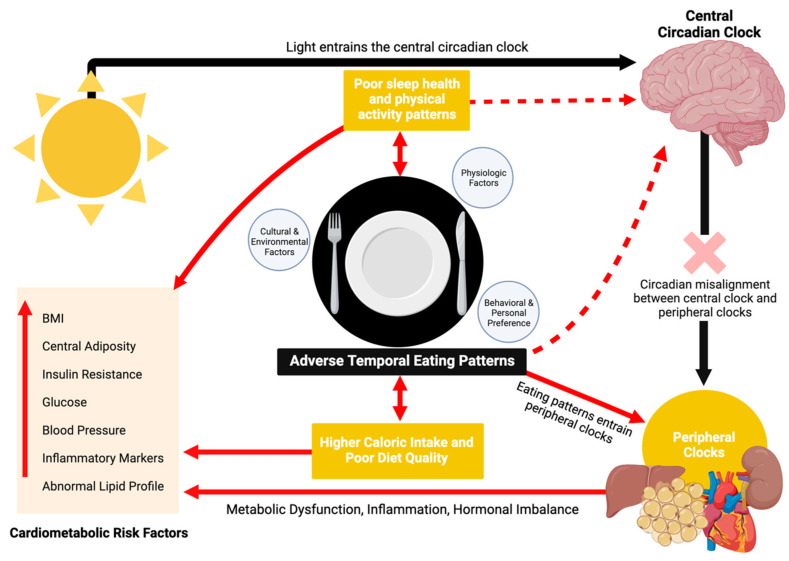
Mechanisms linking temporal eating patterns to cardiometabolic disease. Created with BioRender.com (accessed on 19 December 2023).

The influence of eating timing on CMH operates through various mechanisms related to circadian rhythm disruption, metabolic and inflammatory processes, hormonal imbalances, and other behavioral factors (Figure 2) [3,31,32]. Although the rhythm of the central circadian clock (located in the suprachiasmatic nucleus of the hypothalamus) aligns predominantly with photic cues, the rhythms of peripheral tissues are primarily governed by external cues such as food intake, a powerful zeitgeber [33,34]. Adverse temporal eating patterns, characterized by delayed and irregular eating timing, can disrupt the synchrony of these peripheral clocks with the central clock, resulting in circadian misalignment [34]. Circadian misalignment disrupts metabolic regulation or homeostasis, resulting in impaired glucose control, increased insulin levels, insulin resistance, and a glucose response that mimics a prediabetic state [35]. Notably, late eating has been shown to decrease glucose tolerance and impair pancreatic beta cell function in individuals carrying the MTNR1B type 2 diabetes risk variant [36]. Eating timing also entrains blood pressure (BP) levels and circadian patterns [37]. Short-term circadian misalignment has been shown to elevate BP by modulating the autonomic nervous system, reducing sympathetic activity while increasing parasympathetic activity. Circadian misalignment also diminishes the secretion of melatonin, which is known to lower BP [38]. Additionally, later and irregular eating timing patterns are associated with higher C-reactive protein (CRP), a marker of systemic inflammation linked to high BP and other cardiovascular complications [32,39]. 

Later eating timing and circadian misalignment from mismatched eating patterns and endogenous rhythms can also alter appetite-regulating hormones, increasing the risk of obesity and its metabolic sequelae [35]. For example, inverting behavioral cycles by ~12 h can suppress leptin and lead to increased appetite and decreased energy expenditure. Overall, later eating timing alters appetite-regulating hormones by reducing 24 h serum leptin and increasing the 24 h ghrelin–leptin ratio, promoting increased hunger [40]. It is also associated with decreased energy expenditure during the day and changes in gene expression that favor increased lipid storage and promote obesity [40]. Lastly, the timing of high-energy meals can impact fat accumulation and metabolism through the adipose tissue clock, potentially contributing to dyslipidemia and obesity [41].

Other potential mechanisms requiring further investigation include the impact of eating timing on the gut microbiome and gene expression. Alterations in the composition, function, and rhythms of the human gastrointestinal microbiota, as well as changes in clock and metabolic gene expression, have been reported in response to eating timing cues in emerging studies [42,43]. For example, later eating timing has been shown to induce changes in the microbiota similar to those observed in women with obesity or in older age [44]. In terms of the interplay of eating timing with genetic factors, the PERIOD2 (PER2) genes have been associated with Night Eating Syndrome in prior research [45]. In addition, the minor allele (G) of the CLOCK rs4580704 SNP, associated with obesity, has been shown to be more prevalent among late eaters (after 3:00 pm) compared to early eaters (before 3:00 pm) and has been linked to consumption of a later lunch [17]. Indeed, it appears that the heritability of food timing may vary by meal; a Spanish study of twins showed that it ranged from 56% for breakfast, 38% for lunch, to being undetectable for dinner [46]. Another study of twin pairs showed ~24% heritability for the timing of breakfast, and lower heritability estimates for lunch and dinner timing (18–22%) [47]. There is also evidence of a potential shared genetic architecture between food timing and chronotype that warrants further investigation [46,47].

There is likely a bidirectional association between eating timing and mental health, which can influence eating habits and the quality of food intake [48,49,50]. Shift workers, who often experience misalignment between their internal circadian rhythms and daily eating schedules, are at increased risk of depression and anxiety [49,51]. Consuming meals during daytime hours versus during both the daytime and nighttime has been shown to reduce levels of depression-like and anxiety-like moods [52]. Furthermore, the timing and regularity of eating patterns may influence other health behaviors that impact CMH [53,54,55,56]. For example, both skipping breakfast and irregular eating patterns have been linked to lower sleep quality and reduced physical activity [55,57]. Lastly, eating timing may influence diet quality; individuals who consume food during the nighttime tend to have a higher energy intake from snacks, a greater percentage of their daily energy intake coming from fats, and lower dietary diversity scores [58].

## 4. Summary of Observational Evidence

### 4.1. Breakfast Skipping and Timing of First Eating Occasion

In the prospective Health Professionals Follow-up Study (N = 26,902), men who omitted breakfast exhibited a 27% elevated coronary heart disease (CHD) risk, and the association was mediated by BMI, hypertension, diabetes, and hypercholesterolemia [59]. Indeed, breakfast skipping has been linked to greater type 2 diabetes risk, higher total and LDL cholesterol levels, body weight, fat mass, and abdominal adiposity and lower HDL cholesterol, though most studies focus on obesity risk [60,61,62]. For instance, a landmark study of >50,000 Seventh-day Adventists in the US and Canada revealed that individuals who consumed their largest meal at breakfast vs. dinner had a significant decrease in their body mass index (BMI) over a 7 year follow-up period [63].

However, there are some contradictory findings to the aforementioned studies. Among Japanese adults without obesity, breakfast skipping was associated with 28% and 57% higher odds of developing metabolic syndrome and obesity, respectively, but only when in combination with eating late-night dinners (within 2 h of bedtime) [11]. A cross-sectional analysis of healthy Korean adults revealed that those who infrequently ate breakfast had lower odds of having elevated serum triglycerides compared to those who regularly or frequently consumed breakfast [64]. This could be explained by the larger proportion of energy intake from fats and smaller proportion from carbohydrates observed among the infrequent breakfast eaters.

In addition to breakfast consumption, the timing of the first eating occasion has also been linked to CMH [65,66]. Morning eating, regardless of whether or not the meal is identified as breakfast and independent of fasting duration and number of eating episodes, has been linked to 27% and 31% lower odds of metabolic syndrome in Asian men and women, respectively [12]. Among US women encompassing different life stages, later timing of the first eating occasion was associated with poorer cardiovascular health, defined using the AHA’s Life’s Simple 7 score, as well as with higher waist circumference, diastolic BP, and fasting glucose levels [65]. Conversely, a prospective study conducted with older adults revealed that a timing of first eating occasion after 09:00 am might be linked to a reduced long-term risk of developing type 2 diabetes, particularly among older individuals with impaired fasting glucose, suggesting that associations may vary by life stage and chronic disease status [66].

Only one prospective cohort investigated eating timing patterns in relation to incident CVD [67]. In a 2023 analysis that encompassed >100,000 French adults who participated in the NutriNet-Santé cohort, each 1 h delay in timing of the first meal was associated with 6% higher risk of overall CVD [67]. Notably, every 1 h increase in nightly fasting duration in that study predicted a 7% lower risk of cerebrovascular diseases, suggesting that there may be an interplay between eating timing and the span of the daily eating period. In addition, associations were stronger among women, particularly for the timing of the first meal whereby an eating start time later than 9 am was associated with up to a 35% higher risk of CVD and cerebrovascular diseases. The authors attribute these sex differences to the robust associations between temporal eating patterns and cardiometabolic health among women as reported in the aforementioned studies, and also to potential sexual dimorphisms in the anatomy and physiology of the circadian system [67,68,69,70].

### 4.2. Early Lunch vs. Late Lunch

In a post hoc analysis of 420 Mediterranean adults with overweight and obesity who were followed-up for 20 weeks while undergoing a weight loss intervention, consuming lunch earlier in the day (before vs. after 3:00 pm) was associated with pronounced weight loss and lower HOMA-IR, even when caloric consumption and energy expenditure were held constant across both groups [17]. Weight outcomes were not significantly affected by breakfast or dinner times, highlighting the potential health benefits of the Mediterranean diet’s emphasis on lunch as a principal daily meal [17].

### 4.3. Nighttime Eating and Evening Caloric Intake

In a 16-year prospective study of 26,902 middle-aged male health professionals, a 55% higher coronary heart disease (CHD) risk was observed among those who ate late at night, but this was defined as eating after going to bed and does not capture the association of late dinner or snacking with cardiovascular risk [59]. The association was mediated by cardiometabolic risk factors, and several studies have since linked evening caloric intake or late-night eating with cardiometabolic outcomes [59]. 

In the Korean NHANES dataset, night eating, defined as eating after 9:00 pm, was associated with 48% higher odds of metabolic syndrome in men only, suggesting that there may be sex differences in these associations [12]. However, higher nighttime eating levels have been linked to CMH in women as well [39,65]. For instance, each 10% increase in calorie intake between 5:00 pm and midnight was associated with 3% higher CRP levels among 2650 women from NHANES [39]. Consistent with these findings, a greater proportion of daily calories consumed at the self-identified largest evening meal (“dinner” and/or “supper” in an Automated Self-Administered 24 h (ASA24) dietary record) was associated with higher BP in a community-based cohort of 116 New York women [65]. In contrast, an analysis using the NHANES-III dataset (N = 18,407) found an inverse relationship between nighttime eating, defined as consuming ≥25% of daily calories after 7:00 pm, and BMI, even after adjusting for total caloric intake, with nighttime eaters having a predicted BMI 0.44 units lower than those who were not significant nighttime eaters [20]. However, because this analysis relied on a 24 h recall, it may not capture associations of habitual nighttime eating with BMI.

In ~900 middle-aged to older adults, a higher % of daily energy consumed at night (within 2 h before bed time) was associated with 82% higher odds of having overweight and obesity, and ~5 fold higher odds were observed in those with later chronotypes [71]. In contrast, a higher % of daily energy intake consumed during the morning window (within 2 h after wake time) was associated with 47% lower odds of having overweight or obesity, particularly among those with an earlier chronotype [71]. These data suggest that lower energy intake after waking up and higher intakes before bedtime are associated with higher BMI and highlights that associations between eating timing and adiposity likely vary by chronotype. 

Finally, in the NutriNet-Santé study, later timing of the last meal was also associated with an elevated CVD risk, particularly among women [67]. In that study, every 1 h increment in timing of the last meal was associated with an 8% higher CVD risk. A last meal timing between 8:00 p.m. and 9:00 p.m. and after 9:00 p.m. was associated with a 19% and 28% higher CVD risk, respectively. Similar to the results for the timing of the first meal in that study, the associations of last meal timing with incident CVD and cerebrovascular disease were stronger among women. Further, the time interval between the last meal and bedtime, as a proxy for the circadian timing of food intake, was inversely associated with the risk of overall CVD [67].

### 4.4. Eating Timing Variability and Eating Jetlag

An aspect of chrononutrition that is in the nascent stages of characterization is the role of variability in daily eating timing patterns in CMH. In a longitudinal study of racially and ethnically diverse US women (N = 115) with 7-day food records collected using the NIH’s ASA24, significant associations were demonstrated between day-to-day variability of multiple eating timing metrics, captured from the standard deviation of these variables, and eating jetlag, captured from weekday–weekend differences in these metrics, with CMH indicators [22]. Specifically, greater day-to-day variability and eating jetlag in eating start time, span of the daily eating period, and evening caloric intake (% calories consumed after 5:00 p.m. and 8:00 p.m.) were associated with clinically meaningful increases in BMI, waist circumference, HbA1c, and BP [22]. In a subsequent analysis, every 30 min difference in weekday–weekend eating end time was related to a 13% higher CRP level, and every 1 h weekday–weekend difference in eating duration was associated with a 45% elevation in CRP [32]. Consistent with these findings, a positive association between eating jetlag and BMI, independent of chronotype and social jet lag, was observed in a Spanish study of >1000 students; there was a threshold effect, whereby >3.5 h of eating jetlag was associated with a 1.34 kg/m^2^ higher BMI [21]. Notably, eating jetlag in this study was captured by the weekday–weekend difference in eating midpoint and was primarily driven by the later timing of breakfast on weekends compared to weekdays.

## 5. Summary of Intervention Studies

### 5.1. Meal Skipping

Evidence from randomized controlled trials (RCT) addressing the impact of breakfast skipping or not eating before noon on body weight and adiposity metrics is mixed, but it is suggestive of a possible adverse effect on glycemic regulation and blood lipids [55,72,73,74,75,76,77,78]. In a multisite, 16-week, 3-parallel-arm RCT of otherwise healthy adults with overweight and obesity (N = 309), breakfast skipping had no impact on weight loss [72]. Despite this, compliance to the intervention was >90%, suggesting that recommendations to adjust eating timing may be effective at changing eating habits in free-living settings. Comparable outcomes were observed in smaller trials [55,73]. In lean adults (N = 33), no impact was observed on weight loss, but those who ate breakfast had a 10% increase in adipose tissue insulin sensitivity [73]. Similarly, in adults with obesity (N = 23), there was no significant difference in body weight changes between a breakfast consuming group (≥700 kcal before 11:00 a.m. daily, with ~50% consumed within 2 h of waking up) and a breakfast skipping group (abstained from any caloric intake until noon), but the regular omission of breakfast was shown to reduce insulin sensitivity [55]. In a crossover trial (N = 17) that utilized three isocaloric 24 h interventions (comprising a breakfast-skipping day, a dinner-skipping day, and a conventional three-meal-structure day), HOMA-IR and glucose concentrations after lunch were significantly higher after breakfast skipping than dinner skipping [74]. Skipping breakfast also resulted in increased fat oxidation and an intensified inflammatory response in peripheral blood cells after lunch, suggesting that foregoing breakfast can lead to chronic low-grade inflammation and glycemic dysregulation [74].

Despite the null findings for weight loss in the aforementioned studies, in a crossover trial of young adult Japanese males (N = 10), skipping breakfast actually led to a modest yet significant weight gain, and beginning on the sixth day of breakfast omission, an elevated mean 24 h blood glucose was observed [75]. Similarly, a parallel arm RCT (N = 36) found that the breakfast skipping group (control) exhibited a more substantial decrease in weight loss compared to the two breakfast intervention groups but also showed a significant elevation in total cholesterol concentrations [76]. This is consistent with a prior study showing that breakfast omission is linked to increased fasting total and LDL cholesterol levels in healthy lean women [77]. In contrast, a crossover study of 22 patients with diabetes, which compared a meal schedule comprising breakfast, lunch, and dinner to one that skipped breakfast, revealed that individuals who skipped breakfast had significant weight loss but experienced a greater glycemic response after lunch and dinner and had impaired insulin responses to meals [78]. Taken together, these data suggest that the impact of breakfast skipping on weight may vary by chronic disease status, but that breakfast skipping is consistently related to poorer glycemic control.

### 5.2. Eating Timing

In a meta-analysis of twelve RCTs on time-restricted eating, encompassing 730 adults with obesity or overweight, although both early and late time-restricted eating resulted in moderate reductions in body weight and insulin resistance, early time-restricted eating was more effective in improving insulin resistance [8]. However, there were no significant differences in the effects of early vs. later time-restricted eating on fasting blood glucose, BP, and lipid profiles. On the other hand, a systematic review and meta-analysis of nine RCTs that examined the impact of eating timing, in the context of an energy reduced diet, showed that an energy intake distribution that favors the early vs. late timing of food intake is associated with greater weight loss (~1.23 kg) and with lower fasting glucose, insulin resistance, and LDL cholesterol [79].

Earlier timing of the largest meal may be particularly relevant for improving CMH; an RCT of 93 women with overweight and obesity that investigated the effect of a 12-week calorie restricted diet (~1400 kcal/day) with high caloric intake (~50% of total kcal) consumed at breakfast versus dinner showed that the breakfast group had greater weight loss outcomes, including reduced waist measurements, more favorable glucose and lipid profiles, and experienced longer-lasting satiety [80]. In a similar RCT of 60 women with polycystic ovary syndrome, the breakfast group exhibited a 7% and 54% reduction in glucose and insulin levels, respectively, while no association was observed in the dinner group [81]. A randomized crossover trial of 32 women comparing late lunch (at 4:30 pm) to early lunch (at 1:00 pm) reported a 46% increase in glucose levels above the baseline suggesting that a later lunch may reduce glucose tolerance [82]. 

Lastly, an experimental study of female students (N = 12), found that consuming supper at a regular time (6:00 p.m.) versus later (11:00 p.m.) was associated with less efficient digestion and the absorption of carbohydrates at breakfast [83]. This increased efficiency in the late supper group could be attributed to the extended orocecal transit time of the chyme following a late supper, and resulted in elevated blood glucose levels following breakfast the subsequent day [83].

## 6. Research Gaps, Challenges, and Limitations 

A major obstacle to accurately interpreting the evidence on eating timing and regularity in relation to CMH is the heterogeneity in how eating timing, regularity, and the extent of evening eating are defined and measured, making it challenging to compare findings across both observational and intervention studies. For instance, some studies evaluate eating timing (e.g., timing of last eating occasion) while others evaluate meal timing (e.g., timing of self-defined supper or dinner). Similarly, studies evaluating the distribution of caloric intake across the 24 h day may examine the distribution of calories across time bins or meals, at the largest self-identified evening meal (e.g., dinner) or past certain clock times (e.g., % kcal consumed after 5:00 pm or 8:00 pm). In studies of eating regularity, the standard deviation of multiple eating timing metrics has been used to assess day-to-day variability [32], while weekday–weekend differences have been used to capture eating jetlag [21]. Collectively, these variables measure distinct chrononutrition concepts, limiting the ability to draw definitive conclusions and make public health recommendations.

Another important aspect to consider is the methodologies used to assess temporal eating patterns. First, most population-based cohort studies use food frequency questionnaires that lack time-stamped diet data, making the chrononutrition dimension of diet difficult to integrate into observational studies of diet and CMH. Second, most studies rely on self-report, which limits the number of days of diet assessment due to participant burden considerations and introduces measurement error. Studies typically rely on a limited number of 24 h recalls, often collected on non-consecutive days or at a single time point, which are not necessarily representative of habitual intakes. Low-cost, validated diet assessment methods with acceptable participant burden are needed to implement chrononutrition metrics measurement in population studies, enable cross-study comparisons, and support the meaningful translation of research on temporal eating patterns into interventions and public health guidelines aimed at promoting optimal CMH.

Although there is promising emerging evidence from intervention studies, which allow the establishment of causality, regarding the CMH benefits of early and regular eating schedules, modest sample sizes and short follow-up durations are notable limitations. Further, the recruitment of racially and ethnically diverse samples would enhance the generalizability of findings. These methodological limitations, coupled with the inconsistent findings across the heterogenous intervention protocols and the limited data on underlying physiological mechanisms, are barriers to making evidence-based public health guidelines related to eating timing and regularity.

## 7. Research Priorities and Future Research Directions

To advance this field, longitudinal population-based cohort studies of racially and ethnically diverse participants encompassing different life stages are needed to evaluate chrononutrition metrics in relation to healthy longevity. The first step to achieve this is the development of innovative chrononutrition assessment tools that are easily administered in research and public health settings. Ideally, the assessment of temporal eating patterns would span 7–14 consecutive days, but at minimum should capture two weekdays and one weekend day to estimate habitual eating timing and regularity metrics. In the meantime, a promising existing tool is the NIH’s ASA-24, which enables the collection of multiple, automatically coded, self-administered electronic food records and 24 h recalls, as well as sleep data using the novel sleep module [84]. Another convenient tool is the novel “Chrononutrition Questionnaire for General and Shift Work Populations”, a 10–15 min questionnaire for obtaining information on shift work schedules, eating timing, regularity, and frequency, wake and sleep times, and chronotype [85]. These tools enable the investigation of the interplay between eating timing and regularity, multidimensional sleep health, and chronotype, as well as the circadian timing of food intake (i.e., eating timing relative to biological time vs. societal clock time) in relation to CMH, which are all key knowledge gaps in this field. Alternatively, novel tools such as picture-based smartphone applications or wearable technologies that detect eating timing could be developed and validated. In addition, a scientific consensus framework for chrononutrition metrics, akin to the multidimensional sleep health framework [86], should be developed to adequately and holistically capture the impact of dietary rhythms on health. Such a framework could encompass eating duration, eating timing, and eating regularity metrics. 

Notably, most observational and intervention studies focus on obesity risk and weight loss as outcomes [60]; examining other aspects of CMH as outcomes and elucidating underlying physiological mechanisms and biological pathways is another important research direction. In addition, evaluating temporal eating patterns in relation to diurnal patterns of cardiometabolic markers (e.g., incorporating ambulatory BP monitoring and continuous glucose monitoring) may elucidate novel targets for improving CMH using chrononutrition approaches. Importantly, siloed approaches to research on sleep, circadian rhythms, and diet should be avoided. Future observational studies should evaluate the interplay of chrononutrition metrics, sleep and circadian health, and chronotype in CMH. On the other hand, future intervention studies should consider tailoring eating schedules to chronotype and addressing chrononutrition metrics as part of comprehensive multidimensional dietary or circadian health interventions and alongside sleep interventions for optimizing health impact. Insights from behavioral, dissemination, and implementation sciences should be leveraged to design feasible interventions that work in real-world settings and promote adherence. This includes research to understand how wearables, smartphone apps, daily reminders or nudges, educational content, social support, and working with a health professional or peer health educator could enhance participation, engagement, and retention in such studies.

**Figure 3:**
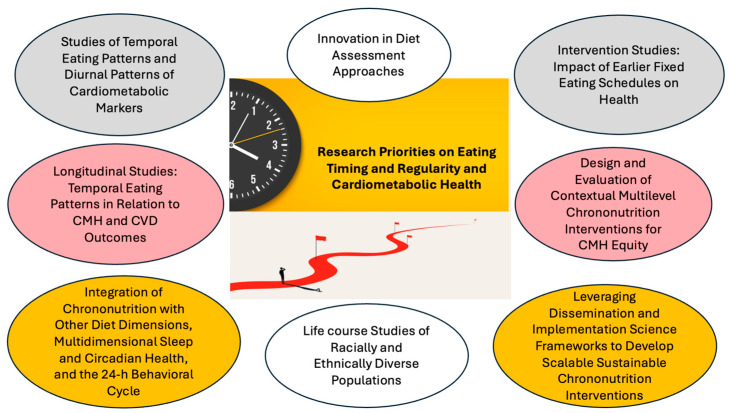
Summary of research priorities on eating timing and regularity and cardiometabolic health.

Given that behavioral and endogenous circadian rhythms vary across life stages and the robustness of circadian rhythms tends to dampen with aging [87,88], adopting a life course approach in future research is crucial to understanding how eating timing and regularity impact CMH preservation, cardiovascular resilience, and healthy longevity. Finally, little is known about the social determinants of health in relation to chrononutrition metrics and how temporal eating patterns contribute to CMH inequities [89]. Chrononutrition metrics are intertwined with sleep and circadian health, which are known to be significant contributors to racial and ethnic disparities in CMH [90,91,92], suggesting that chrononutrition approaches may represent a novel and timely opportunity to promote CMH equity. The research priorities necessary to advance this field and facilitate the translation of chrononutrition research to clinical practice and public health guidelines are summarized in Figure 3.

## 8. Implications for Clinical Practice and Public Health Guidelines and Policy

Upon addressing the aforementioned research gaps, the field of chrononutrition holds great promise for advancing health equity and improving CMH at the individual and population level. At the individual level, personalized chrononutrition interventions addressing the circadian timing of food intake, i.e., eating timing relative to an individual’s biological clock, chronotype, and circadian rhythm, could improve CMH. In clinical and research settings, this approach would involve prescribing a fixed eating schedule customized based on dim light melatonin onset or relative to sleep timing, as a less expensive, more feasible proxy. Importantly, such interventions should be contextual, and multiple levels of influence should be addressed to maximize health benefits. For example, a chrononutrition intervention that also addresses food insecurity would be necessary to ensure consistent mealtimes. The integration of chrononutrition interventions with other lifestyle approaches, such as interventions aimed at improving diet quality or promoting sleep health, could also maximize the health benefit of such interventions. 

At the population level, the integration of the concept of “timing” into public health guidelines would enhance the effectiveness of lifestyle approaches for improving health. Diet guidelines could encourage keeping a consistent eating start time and eating end time each day and recommend that the timing of the last meal occurs before a certain clock time or within a specific interval prior to bedtime. However, additional questions need to be addressed before such quantitative public health recommendations can be made. For example, by how many hours or minutes can eating timing vary day to day before we start observing adverse health effects? Is there a clock time after which eating has adverse health effects for the majority of the population? When should the last eating occasion occur relative to bedtime for optimal health? Similar to individual-level interventions, community-level and population-level chrononutrition interventions should be contextual, addressing social determinants of health and other domains of influence (Figure 1). Implementation science frameworks should be leveraged and advanced to develop culturally responsive, sustainable, and scalable chrononutrition interventions that promote health equity [93].

## 9. Conclusions

Observational and intervention studies suggest that earlier and more regular eating timing patterns may promote CMH, but many knowledge gaps remain to be addressed before these aspects of chrononutrition can be formally integrated into public health guidelines and approaches for chronic disease prevention (Table 1). Currently, it is known that an unhealthy diet is the leading cause of death in the US, particularly from cardiovascular and cardiometabolic disease, but these estimates are based on solely on what (not when) people eat [94]. Elucidating the impact of chrononutrition dimensions on health will be key for quantifying the true impact of multidimensional diet on the chronic disease burden and unlocking the full potential of dietary approaches for addressing persistent CMH inequities by designing and implementing flexible and feasible interventions in real-world settings.

## Figures and Tables

**Table 1:** Summary of key research findings and future research directions.

Chrono-Nutrition Metrics	Key Findings	Research Limitations and Future Directions
Breakfast Consumption	Breakfast skipping generally associated with higher risk of coronary heart disease, type 2 diabetes (higher glucose, insulin, and insulin resistance), hypertension, obesity (higher BMI and central adiposity), and hypercholesterolemia (higher total and LDL cholesterol levels and decreased HDL-cholesterol levels) [59,60,61,62,63]. Mixed evidence on weight, ranging from no impact of breakfast skipping on weight [55,72,73] to breakfast skipping leading to weight gain [75,76] or loss [78]. Breakfast skipping may have an adverse effect on glycemic markers and insulin resistance [64,74,78].	Observational data limitations: Cross-sectional design of most studies. Limited number of studies per chrono-nutrition metric. Reliance on few non-consecutive or single 24 h recalls to assess habitual eating timing patterns. Heterogenous definitions of eating timing and regularity metrics. Residual confounding. Lack of racially and ethnically diverse cohort studies encompassing participants from different life stages. RCT data limitations: Small sample sizes and homogenous study populations. Limited generalizability. Short follow-up duration. Focus on weight loss as an outcome. Future Research Directions: Development of innovative dietary assessment tools that are validated, low cost, and readily administered. Evaluating eating timing and regularity in relation to novel outcomes (e.g., glycemic variability and BP diurnal pattern). Identifying modifiable multi-level determinants of temporal eating patterns (e.g., environmental, sociocultural, and psychosocial factors) and designing multi-level contextual chrono-nutrition interventions for healthy longevity. Investigating the interplay between eating timing and regularity, multidimensional sleep health, and chronotype in cardiometabolic health preservation. Understanding the association of circadian timing of food intake, i.e., eating timing relative to biological timing (captured from dim light melatonin onset or using sleep timing as a practical proxy), in relation to cardiometabolic outcomes. Investigating the role of chrono-nutrition in achieving cardiometabolic health equity. Elucidating novel underlying physiological mechanisms.
Timing of Breakfast or First Eating Occasion	Morning eating is associated with lower odds of metabolic syndrome, while later timing of first eating occasion is linked to poorer overall cardiovascular health, central adiposity, higher BP, and poorer glycemic control [8,12,65,79]. Earlier timing of meals is associated with lower CVD risk, particularly among women [65,67,80,81].	
Timing of Lunch	Earlier timing of lunch linked to greater weight loss, better glycemic control, and lower insulin resistance [17,82].	
Timing of Dinner or Last Eating Occasion and Extent of Evening Caloric Intake	Later eating timing and greater caloric intake in the evening linked to higher cardiovascular disease risk, metabolic syndrome, systemic inflammation, poorer glycemic control, and higher BP [59,67,71,83].	
Eating Regularity and Eating Jetlag	Increased day-to-day variability and weekday–weekend differences in timing of the first and last eating occasion, span of the daily eating period, and evening caloric intake is associated with higher BMI, waist circumference, BP, HbA1c, and CRP [21,22,32].	
